# Enhancing hepatocellular carcinoma management: prognostic value of integrated CCL17, CCR4, CD73, and HHLA2 expression analysis

**DOI:** 10.1007/s00432-024-05832-0

**Published:** 2024-06-25

**Authors:** Wei Gan, Bao-Ye Sun, Zhang-Fu Yang, Cheng Ye, Zhu-Tao Wang, Cheng Zhou, Guo-Qiang Sun, Yong Yi, Shuang-Jian Qiu

**Affiliations:** 1 Department of Pancreatic Surgery, Zhongshan Hospital, Fudan University, Shanghai, China; 2 Department of Liver Surgery and Transplantation & Key Laboratory of Carcinogenesis and Cancer Invasion (Ministry of Education), Zhongshan Hospital, Liver Cancer Institute, Fudan University, Shanghai, China; 3 Department of Otolaryngology, First Affiliated Hospital of Wenzhou Medical University, Wenzhou Medical University, Wenzhou, China

**Keywords:** Hepatocellular carcinoma, Tumor microenvironment, CCL17, CCR4, CD73, Nomogram

## Abstract

**Purpose:**

Hepatocellular carcinoma (HCC) is a critical global health concern, with existing treatments benefiting only a minority of patients. Recent findings implicate the chemokine ligand 17 (CCL17) and its receptor CCR4 as pivotal players in the tumor microenvironment (TME) of various cancers. This investigation aims to delineate the roles of CCL17 and CCR4 in modulating the tumor’s immune landscape, assessing their potential as therapeutic interventions and prognostic markers in HCC.

**Methods:**

873 HCC patients post-radical surgery from 2008 to 2012 at Zhongshan Hospital, Fudan University were retrospectively examined. These individuals were stratified into a training cohort (*n* = 354) and a validation cohort (*n* = 519). Through immunohistochemical analysis on HCC tissue arrays, the expressions of CCL17, CCR4, CD73, CD47, HHLA2, and PD-L1 were quantified. Survival metrics were analyzed using the Cox model, and a prognostic nomogram was devised via R software.

**Results:**

The investigation confirmed the presence of CCL17 and CCR4 within the cancerous and stromal compartments of HCC tissues, associating their heightened expression with adverse clinical markers and survival outcomes. Notably, the interplay between CD73 and CCR4 expression in tumor stroma highlighted a novel cellular entity, CCR4 + CD73 + stromal cells, impacting overall and relapse-free survival. A prognostic nomogram amalgamating these immunological markers and clinical variables was established, offering refined prognostic insights and aiding in the management of HCC. The findings suggest that reduced CCR4 and CCR4 + CD73 + cell prevalence may forecast improved outcomes post-TACE.

**Conclusion:**

This comprehensive evaluation of CCR4, CCL17, and associated markers introduces a nuanced understanding of the HCC immunological milieu, proposing CCR4 + CD73 + stromal cells as critical to HCC pathogenesis and patient stratification.

**Supplementary Information:**

The online version contains supplementary material available at 10.1007/s00432-024-05832-0.

## Introduction

Primary liver cancer ranks fourth globally in cancer-related mortality, with hepatocellular carcinoma (HCC) being the predominant histologic type, constituting 75-85% of cases (Kotsari et al. 2023; Sung et al. 2021; Zhou et al. 2023). Unlike other cancers experiencing a decrease in disease burden, the incidence of HCC continues to rise, and its 5-year survival rate stands at a concerning 12% (Tang et al. 2018; Yang et al. 2019; Zheng et al. 2018). The prognosis of HCC mainly depends on the tumor stage at the time of treatment and the extent of liver function impairment (Gan et al. 2018; Llovet et al. 2021). Patients diagnosed in the early stage may achieve a 5-year survival rate of nearly 70%, while those in advanced stages face a median survival of only 1–2 years (Bangaru et al. 2020). Therefore, there is urgent need to identify and develop more effective therapeutic targets.

Tumors are strongly influenced by the tumor microenvironment (TME), a concept first proposed by Ioannides in 1993, specifically referring to the local environment where tumors develop (Ioannides and Whiteside 1993). TME comprises tumor cells, tumor stromal cells, and non-cellular components of the extracellular matrix (Baghban et al. 2020; Feng et al. 2022). The cells most prominently contributing to HCC progression include regulatory T cells (Tregs), tumor-associated neutrophils (TANs), and tumor-associated macrophages (TAMsWang et al. 2020b; Zheng et al. 2023). Generally, high infiltration of Tregs into tumor tissues correlates with poor prognosis in HCC patients, as well as in many other cancers (Langhans et al. 2019).

Recently, there has been increased focus on chemokine ligands such as Chemokine ligand 17 (CCL17), for which the targeted drug mogamulizumab has been developed(Maeda et al. 2019a). Previous research has shown that aberrantly high expression of CCL17 in various cancers, including leukemia, lung cancer, bladder cancer, breast cancer, gastric cancer, and liver cancer (Bouchet et al. 2020; Gao et al. 2020; Higuchi et al. 2019; Lim et al. 2014; Mishalian et al. 2014; Wang et al. 2018, 2019; Ye et al. 2022), serves as a primary driver for recruiting CCR4-expressing Tregs into the TME. This, in turn, extensively suppresses the anti-tumor immune response and promotes tumor progression(Gao et al. 2020). However, CCL17-positive cells have only been reported to be distributed within the tumor stroma in the context of HCC (Zhou et al. 2016). The direct release of CCL17 by tumor cells has not yet been characterized, and the specific role of CCL17 in the interaction between tumor and tumor stroma in the TME remains undefined. Furthermore, there is no definitive evidence to determine whether CCL17 might be an independent target for cancer therapy or whether it might cooperate with other effective immune checkpoint inhibitors to enhance the anti-tumor effect in HCC. Therefore, the relationship between the expression levels of immune checkpoints and CCL17 in HCC merits further exploration.

Ecto-5’nucleotidase, commonly referred to as CD73, functions as a converter that transforms AMP into extracellular adenosine (Harvey et al. 2020). Previous studies have identified an overexpression of CD73 in many solid tumors, including HCC, and this overexpression is frequently associated with poor prognosis (Harvey et al. 2020; Ma et al. 2020). Additionally, CD73 is linked to tumor metastasis, drug resistance, cancer-stem-cell traits, and has been identified as a novel immune checkpoint (Lupia et al. 2018; Ma et al. 2020). Recent research has shown that CD73 can be expressed by various cell types including immune cells, stromal cells, epithelial cells, endothelial cells, and cancer cells (Da et al. 2022; Yu et al. 2020). However, there remains limited knowledge about the relationship between CD73, CCL17, and CCR4 within the tumor microenvironment in HCC.

Transcatheter arterial chemoembolization (TACE), a treatment that combines chemotherapeutic drug injection with occlusion of the tumor’s blood supply, has emerged as a primary option for patients with BCLC (Barcelona Clinic Liver Cancer) stage B HCC (Dev et al. 2020). The application of TACE, however, is based on traditional tumor characteristics, making it difficult to predict which patients will benefit most from postoperative TACE therapy (Wang et al. 2020a). This complexity highlights the need for reliable indicators for TACE therapy.

This study seeks to investigate the clinical significance of CCR4, CCL17, CD73, and HHLA2 in HCC, exploring their interconnectedness within the tumor immune microenvironment. Additionally, we aim to identify a novel index to predict the effectiveness of postoperative TACE and construct predictive nomograms for overall survival (OS) and recurrence-free survival (RFS), thereby enhancing clinical decision-making in the management of HCC.

## Materials and methods

### Patients and follow-up

A total of 873 consecutive HCC patients who underwent radical operations at Zhongshan Hospital, Fudan University were enrolled. Patients were stratified into two distinct cohorts based on admission time: an early cohort, comprising 354 patients admitted between 2008 and 2009, served as the training group, while a late cohort, including 519 patients admitted between 2010 and 2012, constituted the validation group. The inclusion criteria were as follows: (1) patients with pathologically diagnosed HCC, (2) patients accepting no preoperative treatment, (3) patients with complete tumor excision and negative incisal margins confirmed by pathology, (4) patients without evidence of other malignant tumors, (5) patients with detailed clinicopathological and follow-up data. Appropriate tumor tissues were subjected to formalin-fixing and paraffin-embedding, and two expert pathologists were assigned to confirm the clinical stage. RFS was defined as the period from surgery to the date of confirmed recurrence o, while OS was recorded from the date of surgery to death.

### Tissue microarray (TMA), immunohistochemistry (IHC), and immunofluorescence (IF)

TMAs were prepared from formalin-fixed, paraffin-embedded surgical specimens that were HE-stained to select representative tumor areas. Triplicate cores of 1 mm diameter were taken from each individual tumor.

Experienced pathologists performed the IHC staining for CCL17 (abcam, 2.5 µg/mL), CCR4 (abcam, 5 µg/mL), CD73 (Bio-Techne, 5 µg/mL), CD47 (abcam, 0.3 µg/mL), HHLA2 (Atlas, 0.5 µg/mL), PD-L1 (abcam, 2 µg/mL), CD34 (abcam, 0.22 µg/mL), CD68 (CST, 1:500), CD66b (abcam, 0.026 µg/mL), Foxp3 (abcam, 3.67 µg/mL), CD8 (abcam, 0.4 µg/mL), CD4 (abcam, 1.3 µg/mL). The slides underwent incubation, deparaffinization, rehydration, antigen retrieval (10 min at 120 °C in a citrate buffer, pH 6.0), and endogenous peroxidase inactivation. Then, they were sequentially incubated with primary and secondary antibodies. The preparation for IF was identical to that for IHC, but followed by fluor-conjugated secondary antibody. The experimental procedure of IHC and IF staining of TMA was performed as described in our previous study (Gao et al. 2007; Jing et al. 2019).

### Quantification of CCL17, CCR4, CD73, CD47, HHLA2, PD-L1, and immune cell infiltration

CCL17, CCR4, CD73, CD47, HHLA2, and PD-L1 protein expressions in tumor (marked as CCL17-T, CCR4-T, CD73-T, CD47, HHLA2, and PD-L1) were evaluated by immunohistochemistry and quantified using a combined score (CS: % positive cells × intensity). Specifically, the percentage of positive cells was categorized into four levels: 1 (1-10%), 2 (11-50%), 3 (51-80%), and 4 (> 80%), while staining intensity was graded on a scale of 0 (no staining), 1 (light yellow), 2 (tan), and 3 (dark brown). In the survival analysis, low expression was defined as a CS score of 0–4, while high expression was defined as a score of 5–12. Additionally, we assessed the expression levels of these proteins, including CCL17-I, CCR4-I, and CCR4 + CD73+, within the tumor stroma by quantifying the number of positive cells. Moreover, the numbers of CD8+, CD66+, or FOXP3 + cells were quantified in five high-power fields (400X) for each group. The optimal cutoff values for stromal expression were determined using X-tile analysis, as described in our previous study (Zhang et al. 2018).

### Statistical analysis

All statistical analyses were conducted using SPSS version 21 (IBM Corporation, Armonk, NY, USA). Associations between CCL17, CCR4, CD73, HHLA2, PD-L1, and other variables, such as tumor size and AFP, were assessed using the Pearson chi-squared test. The Kaplan-Meier method was employed to evaluate the survival curves of OS and PFS for patients, while differences between groups were calculated using the log-rank test. Additionally, univariate and multivariate analyses were executed with the Cox proportional hazards model. A new nomogram was constructed using R software version 3.0.2.

## Results

### Patient characteristics

We analyzed TMAs comprising 873 HCC tissues sourced from Zhongshan Hospital, categorizing them into two distinct groups: a training cohort consisting of 354 HCC specimens and a validation cohort composed of 519 HCC cases. It is noteworthy that in our cohorts, the etiology of HCC in China predominantly involves hepatitis B virus (HBV) infection, with an observed infection rate of 87.3%. In the training cohort, the OS rates at 1, 3, and 5 years were observed to be 96%, 79%, and 66%, respectively. Concurrently, the RFS rates for the same time intervals were 87%, 68%, and 56%, respectively. Meanwhile, in the validation cohort, the OS rates at 1, 3, and 5 years were recorded at 91%, 56%, and 40%, respectively, with corresponding RFS rates of 76%, 42%, and 29%. Comparisons of training cohort and validation cohort clinical characteristics are listed in Table 1.

**Table 1:** The comparation between training cohort and validation cohort

Characters	Training cohort	Validation cohort	*p*-value
	(*n* = 354)	(*n* = 519)	
Gender, male/female	301/53	433/86	0.526
Age, < 60/≥60	239/115	284/235	< 0.001
Asite, no/yes	335/19	488/31	0.705
Cirrhosis, no/yes	243/111	285/234	< 0.001
Tumor number, single/multiple	292/62	453/66	0.049
Tumor size, < 5/≥5 cm	210/144	296/223	0.862
Tumor capsule, no/yes	105/249	333/186	< 0.001
Bleeding, no/yes	322/32	475/44	0.773
Differentiation, I-II/III-IV	222/132	341/178	0.364
MVI, no/yes	205/149	381/138	< 0.001
HBsAg, negative/positive	45/309	75/444	0.464
AFP, < 400/≥400 ng/mL	162/192	365/154	< 0.001
TB, < 45/≥45 U/L	307/47	474/45	0.030
GGT, < 45/≥45 U/L	143/211	215/304	0.761
ALT, < 50/≥50 U/L	285/69	417/102	0.953
ALB, < 35/≥35 g/L	27/327	177/342	< 0.001
CD4, < 80/≥80	307/47	440/79	0.422
CD34, Low/high	174/180	240/279	0.398
CD68, < 100/≥100	225/129	324/195	0.734
CD66b, < 22/≥22	224/130	297/222	0.074
Treg/CD8, < 1/≥1	230/124	208/311	< 0.001
Treg, < 19/≥19	214/140	205/314	< 0.001
CD8, < 50/≥50	178/176	180/339	< 0.001
CCR4-T, Low/High	175/179	231/288	0.152
CCR4-I, < 25/≥25	192/162	273/246	0.634
CCL17-T, Low/High	185/169	257/262	0.426
CCL17-I, < 30/≥30	141/213	209/310	0.897
HHLA-2-T, Low/High	199/155	269/250	0.202
CD73-T, Low/High	146/208	192/327	0.206
CCR4 + CD73+, < 13/≥13	170/184	270/249	0.246
CD47, Low/High	226/128	341/178	0.571
PDL1, Low/High	211/143	310/209	0.970

APF, alpha fetal protein; MVI, microvascular invasion

### Tumor expression of CCL17 and CCR4 proteins

**Fig. 1:**
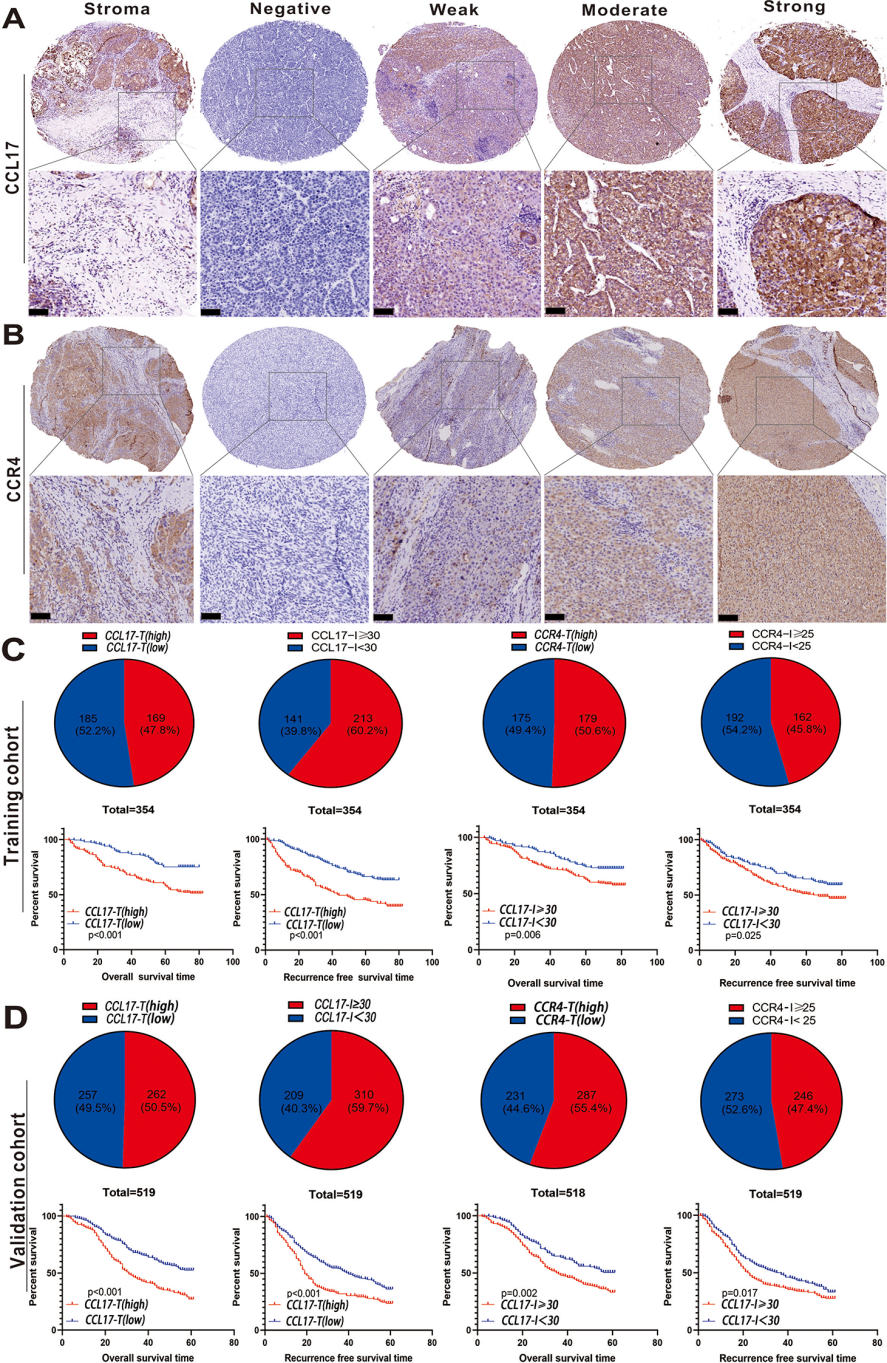
Expression of CCL17 and CCR4 in HCC tissue samples and their impact on prognosis. Representative micrographs of CCL17 (**A**) and CCR4 (**B**) expression within tumor and stroma; Kaplan-Meier survival curves for OS and RFS in HCC patients, high CCL17-T expression and CCL17-I (≥ 30) demonstrated significant associations with poor OS and RFS in the training cohort (**C**) and validation cohort (**D**), respectively. (Scale bar = 50 μm)

To evaluate the expression pattern of CCL17, CCR4 in HCC, we performed IHC on TMAs. Representative microphotographs of CCL17-T and CCR4-T immunostaining are displayed (expressions in tumor). Figure 1A-B shows representative IHC staining of CCL17-T and CCR4-T expressions, categorized as “negative,” “weak,” “moderate,” and “strong,” reflecting ascending levels of expression.

Intriguingly, CCL17 and CCR4 were also found to be widely expressed in tumor stroma, hinting at a subtype of stromal cells, mainly immune cells, expressing these two molecules. This observation aligns with previous reports that identified CCL17 and CCR4 as critical participants in the tumor’s immunosuppressive environment. To further understand CCL17 and CCR4’s role in the tumor microenvironment, expressions in the tumor stroma (CCL17-I and CCR4-I) were also analyzed (Fig. 1A-B).

### High CCL17 and CCR4 expression impaired HCC clinical outcomes

The training cohort was divided into high and low CCL17-T expression groups according to IHC staining results. Out of 354 patients, 169 (47.8%) were classified into the high-expression group, while 185 (52.2%) were assigned to the low-expression group. Notably, increased CCL17-T levels were primarily found in patients with multiple tumor lesions, poor tumor differentiation, and microvascular invasion (MVI). Additionally, a significant correlation was identified between high CCL17-T expression and several prognostic indicators in HCC, including CD34, the Treg/CD8 ratio, HHLA2, and CD73-T (all *P* < 0.050, Table 2). Kaplan-Meier survival analysis indicated that elevated levels of CCL17-T and CCL17-I were significantly associated with reduced OS and RFS in HCC patients (both *P* < 0.050, Table 3; Fig. 1C). In a similar vein, patients with high CCR4-T expression (179, 50.6%) demonstrated markedly shorter OS (*P* < 0.001) and RFS (*P* = 0.039), whereas CCR4-I expression did not exhibit a statistically significant impact on HCC patient outcomes (Table 3). These observations were further corroborated by the validation cohort, emphasizing the importance of these findings for the clinical management of HCC (Table 4; Fig. 1D).

**Table 2:** Demographic and clinical characteristics of patients in training cohort

Characters	Total patients (*n* = 354)	CCL17-T			CCR4-T		
		Low	High	*p*-value	Low	High	*p*-value
Gender, male/female	301/53	161/24	140/29	0.270	148/27	153/26	0.812
Age, < 60/≥60	239/115	125/60	114/55	0.982	117/58	122/57	0.794
Asite, no/yes	335/19	178/7	157/12	0.167	164/11	171/8	0.448
Cirrhosis, no/yes	243/111	135/50	108/61	0.066	112/63	131/48	0.063
Tumor number, single/multiple	292/62	165/20	127/42	0.001	144/31	148/31	0.922
Tumor size, < 5/≥5 cm	210/144	114/71	96/73	0.357	103/72	107/72	0.860
Tumor capsule, no/yes	105/249	51/134	54/115	0.367	48/127	57/122	0.363
Bleeding, no/yes	322/32	169/16	153/16	0.788	155/20	167/12	0.121
Differentiation, I-II/III-IV	222/132	127/58	95/74	0.016	109/66	113/66	0.870
MVI, no/yes	205/149	125/60	80/89	< 0.001	111/64	94/85	0.038
HBsAg, negative/positive	45/309	26/159	19/150	0.428	20/155	25/154	0.474
AFP, < 400/≥400 ng/mL	162/192	91/94	71/98	0.176	82/93	80/99	0.683
TB, < 45/≥45 U/L	307/47	157/28	150/19	0.281	148/27	159/20	0.238
GGT, < 45/≥45 U/L	143/211	82/103	61/108	0.115	63/112	80/99	0.096
ALT, < 50/≥50 U/L	285/69	149/36	136/33	0.987	138/37	147/32	0.438
ALB, < 35/≥35 g/L	27/327	17/168	10/159	0.247	26/149	1/178	< 0.001
CD4, < 80/≥80	307/47	160/25	147/22	0.891	155/20	152/27	0.311
CD34, Low/high	174/180	103/82	71/98	0.010	104/71	70/109	< 0.001
CD68, < 100/≥100	225/129	125/60	100/69	0.101	123/52	102/77	0.009
CD66b, < 22/≥22	224/130	122/63	102/67	0.276	116/59	108/71	0.246
Treg/CD8, < 1/≥1	230/124	136/49	94/75	< 0.001	113/62	117/62	0.876
Treg, < 19/≥19	214/140	115/70	99/70	0.491	108/67	106/73	0.631
CD8, < 50/≥50	178/176	90/95	88/81	0.520	96/79	82/97	0.089
CCR4-T, Low/High	175/179	91/94	84/85	0.923			
CCR4-I, < 25/≥25	192/162	105/80	87/82	0.319	97/78	95/84	0.656
CCL17-I, < 30/≥30	141/213	78/107	63/106	0.348	70/105	71/108	0.949
HHLA2, Low/High	199/155	108/77	81/88	0.049	98/77	91/88	0.330
CD73-T, Low/High	146/208	86/99	60/109	0.047	77/98	69/110	0.297
CCR4 + CD73+, < 13/≥13	170/184	93/92	77/92	0.376	96/79	74/105	0.011
CD47, Low/High	226/128	113/72	113/56	0.258	83/92	143/36	< 0.001
PDL1, Low/High	211/143	106/79	105/64	0.355	106/69	105/74	0.714
CCL17-T, Low/High	185/169				91/84	94/85	0.923

APF, alpha fetal protein; MVI, microvascular invasion

### CD73 and HHLA2 inversely correlated with HCC prognosis

**Fig. 2:**
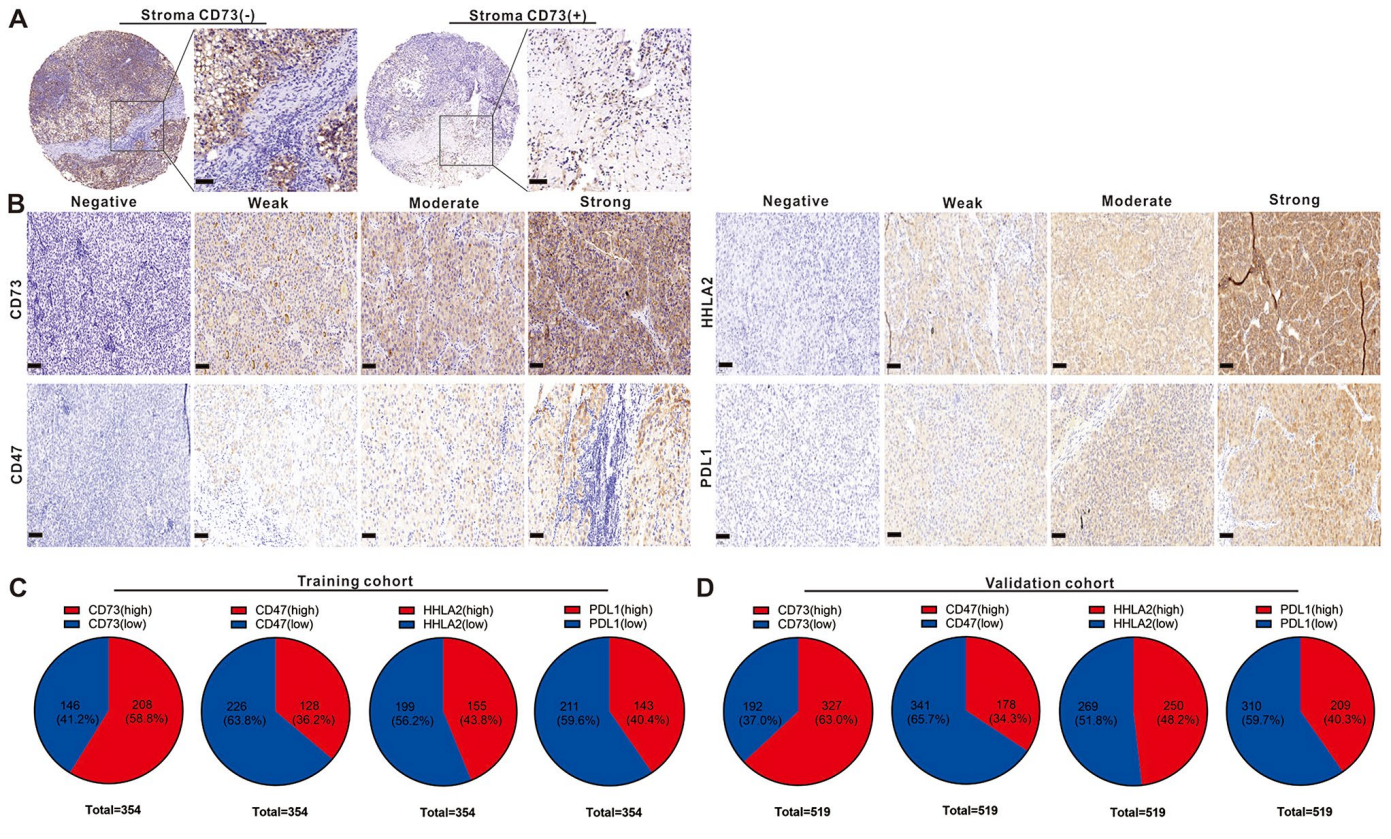
Expression of CD73, CD47, HHLA2, and PDL1 in HCC tissue samples. Representative micrographs illustrating the expression of PDL1, HHLA2, CD47, and CD73 in tumor tissue (**B**) and the positive or negative expression of CD73 in the stroma (**A**). Statistical expression profiles of CD73, CD47, HHLA2, and PDL1 in training (**C**) and validation cohorts (**D**). (Scale bar = 50 μm)

We subsequently explored the associations between immune checkpoints and clinical outcomes in HCC. CD73, CD47, HHLA2, and PDL1 were selected for analysis due to their recognized immunosuppressive role in HCC. Expression levels were assessed through IHC as depicted in Fig. 2A-B. In the training cohort, the rates of high CD73, CD47, HHLA2, and PDL1 expression were 58.8%, 36.2%, 43.8%, and 40.4%, respectively. In the validation cohort, the percentages of high CD73, CD47, HHLA2, and PDL1 expression were 63.0%, 34.3%, 48.2%, and 40.3%, respectively (Fig. 2C-D). Survival analysis demonstrated that high CD73 or HHLA2 expression independently correlated with poor OS and RFS, whereas CD47 and PDL1 did not serve as predictors (Tables 3 and 4).

**Table 3:** Univariate and multivariate analyses for OS and RFS in patients with HCC in training cohort

Characters	Total Patients (*n* = 354)	Univariate		Multivariate					
		OS	RFS	OS			RFS		
		*p*-value	*p*-value	HR	95% CI	*p*-value	HR	95% CI	*p*-value
Gender, male/female	301/53	0.688	0.107			-			-
Age, < 60/≥60	239/115	0.217	0.817			-			-
Asite, no/yes	335/19	0.122	0.775			-			-
Cirrhosis, no/yes	243/111	0.487	0.033			-	1.844	1.293–2.630	0.001
Tumor number, single/multiple	292/62	0.007	0.042			ns.			ns.
Tumor size, < 5/≥5 cm	204/150	< 0.001	0.002	1.824	1.258–2.644	0.002	1.553	1.095–2.203	0.014
Tumor capsule, no/yes	105/249	0.086	0.201			-			-
Bleeding, no/yes	322/32	0.158	0.961			-			-
Differentiation, I-II/III-IV	222/132	0.021	0.025			ns.			ns.
MVI, no/yes	205/149	< 0.001	< 0.001	1.529	1.026–2.277	0.037	1.427	1.005–2.024	0.047
HBsAg, negative/positive	45/309	0.429	0.393			-			-
AFP, < 400/≥400 ng/mL	162/192	0.003	0.018			ns.			ns.
TB, < 45/≥45 U/L	307/47	0.082	0.147			-			-
GGT, < 45/≥45 U/L	143/211	0.024	0.012			ns.			ns.
ALT, < 50/≥50 U/L	285/69	0.489	0.198			-			-
ALB, < 35/≥35 g/L	27/327	0.122	0.94			-			-
CD4, < 80/≥80	307/47	0.419	0.675			-			-
CD34, Low/high	174/180	< 0.001	0.035	1.583	1.085–2.309	0.017			ns.
CD68, < 100/≥100	225/129	< 0.001	0.393	1.488	1.021–2.168	0.039			-
CD66b, < 22/≥22	224/130	0.002	0.337			ns.			-
Treg/CD8, < 1/≥1	230/124	< 0.001	< 0.001	1.532	1.050–2.235	0.027	1.456	1.036–2.046	0.030
Treg, < 19/≥19	214/140	0.323	0.301			-			-
CD8, < 50/≥50	178/176	0.239	0.051			-			-
CCR4-T, Low/High	175/179	< 0.001	0.039	2.158	1.458–3.195	< 0.001	1.556	1.114–2.174	0.010
CCR4-I, < 25/≥25	192/162	0.508	0.160			-			-
CCL17-T, Low/High	185/169	< 0.001	< 0.001	1.818	1.242–2.661	0.002	1.774	1.274–2.470	0.001
CCL17-I, < 30/≥30	141/213	0.006	0.025			ns.			ns.
HHLA2-T, Low/High	199/155	< 0.001	0.007	1.627	1.127–2.347	0.009	1.407	1.011–1.958	0.043
CD73-T, Low/High	146/208	< 0.001	< 0.001	1.891	1.247–2.868	0.003	1.481	1.037–2.115	0.031
CCR4 + CD73+, < 13/≥13	170/184	< 0.001	0.015	1.594	1.082–2.349	0.018	1.486	1.060–2.083	0.022
CD47, Low/High	226/128	0.964	0.283			-			-
PDL1, Low/High	211/143	0.552	0.810			-			-

APF, alpha fetal protein; MVI, microvascular invasion; ns. not significant

**Table 4:** Univariate and multivariate analyses for OS and RFS in patients with HCC in validation cohort

Characters	Total Patients (*n* = 519)	Univariate		Multivariate					
		OS	RFS	OS			RFS		
		*p*-value	*p*-value	HR	95% CI	*p*-value	HR	95% CI	*p*-value
Gender, male/female	433/86	0.466	0.780			-			-
Age, < 60/≥60	284/235	0.039	0.014			ns.	1.419	1.133–1.777	0.002
Asite, no/yes	488/31	0.534	0.496			-			-
Cirrhosis, no/yes	285/234	0.170	0.856			-			-
Tumor number, single/multiple	453/66	0.040	0.002			ns.			ns.
Tumor size, < 5/≥5 cm	296/223	< 0.001	< 0.001	1.352	1.037–1.761	0.026	1.489	1.163–1.906	0.002
Tumor capsule, no/yes	333/186	0.618	0.260			-			-
Bleeding, no/yes	475/44	0.554	0.584			-			-
Differentiation, I-II/III-IV	341/178	0.025	0.048	1.303	1.013–1.677	0.040			ns.
MVI, no/yes	381/138	0.002	< 0.001	1.582	1.203–2.082	0.001	1.799	1.398–2.317	< 0.001
HBsAg, negative/positive	75/444	0.939	0.871			-			-
AFP, < 400/≥400 ng/mL	365/154	0.010	0.132			ns.			-
TB, < 45/≥45 U/L	474/45	0.043	0.595			ns.			-
GGT, < 45/≥45 U/L	215/304	0.030	< 0.001			ns.			ns.
ALT, < 50/≥50 U/L	417/102	0.993	0.025			-			ns.
ALB, < 35/≥35 g/L	177/342	0.003	0.086			ns.			-
CD4, < 80/≥80	440/79	0.939	0.852			-			-
CD34, Low/high	240/279	< 0.001	0.004	1.365	1.062–1.755	0.015			ns.
CD68, < 100/≥100	324/195	0.798	0.321			-			-
CD66b, < 22/≥22	297/222	0.045	0.044			ns.			ns.
Treg/CD8, < 1/≥1	208/311	< 0.001	< 0.001	1.721	1.329–2.228	< 0.001	1.551	1.214–1.983	< 0.001
Treg, < 19/≥19	205/314	0.015	0.048			ns.			ns.
CD8, < 50/≥50	180/339	0.021	0.628			ns.			-
CCR4-T, Low/High	231/288	< 0.001	< 0.001	2.427	1.862–3.163	< 0.001	2.191	1.705–2.816	< 0.001
CCR4-I, < 25/≥25	273/246	0.058	0.051			-			-
CCL17-T, Low/High	257/262	< 0.001	< 0.001	1.633	1.274–2.095	< 0.001	1.407	1.117–1.771	0.004
CCL17-I, < 30/≥30	209/310	0.002	0.017	1.657	1.286–2.136	< 0.001	1.341	1.061–1.695	0.014
HHLA2-T, Low/High	269/250	0.046	0.029	1.372	1.077–1.748	0.010	1.424	1.139–1.781	0.002
CD73-T, Low/High	192/327	< 0.001	< 0.001	1.937	1.474–2.547	< 0.001	1.279	1.004–1.629	0.046
CCR4 + CD73+, < 13/≥13	270/249	0.001	0.015	1.890	1.456–2.455	< 0.001	1.582	1.234–2.028	< 0.001
CD47, Low/High	341/178	0.859	0.216			-			-
PDL1, Low/High	310/209	0.804	0.822			-			-

APF, alpha fetal protein; MVI, microvascular invasion; ns. not significant

### CCL17 and CCR4 interact closely within the tumor microenvironment

**Fig. 3:**
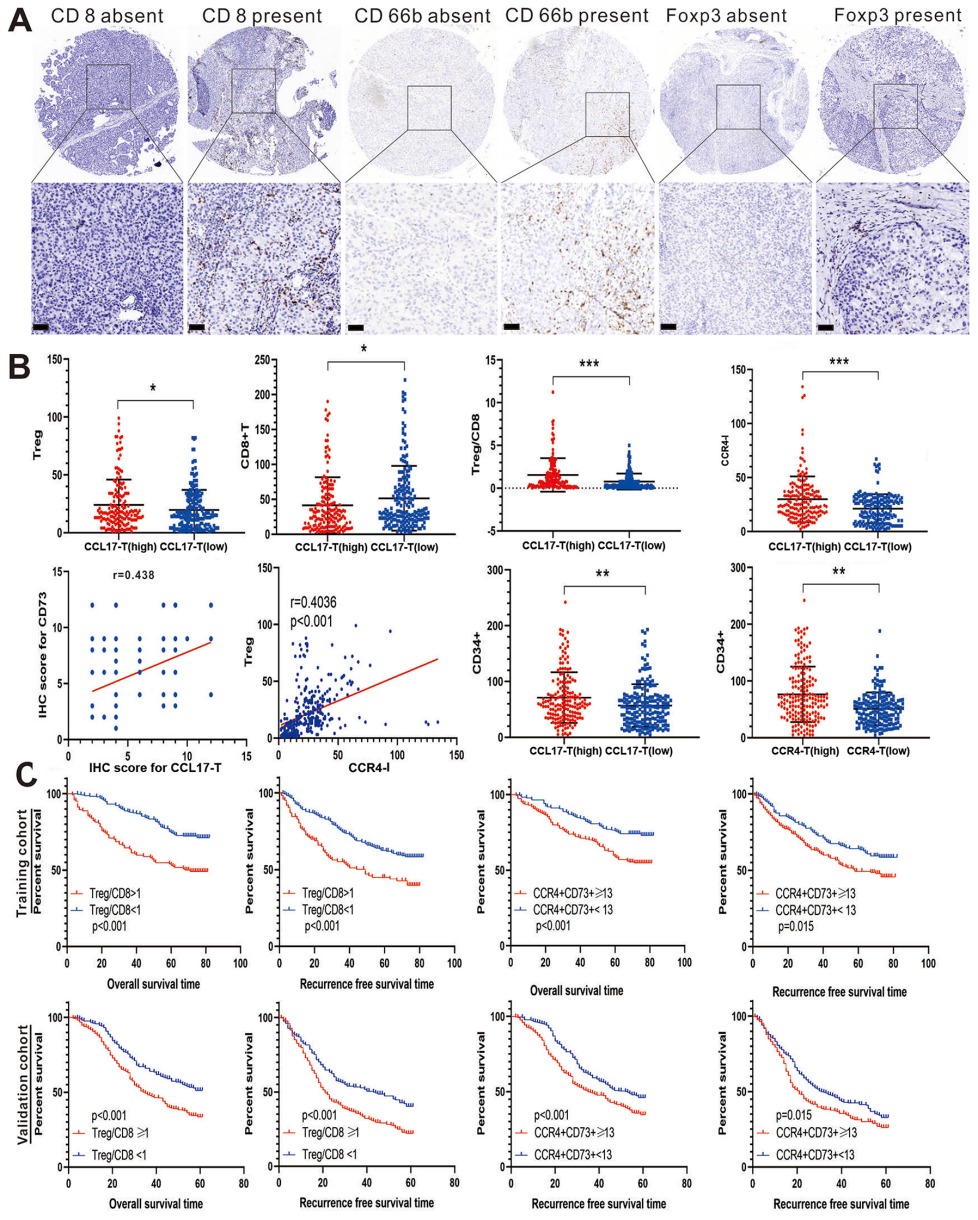
Tumor-infiltrating immune cells and their correlation with CCL17, CCR4, CD73, as well as their impact on prognosis. (**A**) IHC images show positive staining for CD8 (cytotoxic T cells), CD66b (tumor-associated neutrophils), and FOXP3 (regulatory T cells), along with relevant intra-tumor negative controls. (**B**) High CCL17-T expression significantly correlates with increased Tregs, lower CD8 + counts, and the ratio of Tregs to CD8, as well as CCR4-I. The scatter plot indicates a close relationship between CCL17-T and CD73 protein expression. Both high CCL17-T and CCR4-T expression are associated with elevated CD34 + counts. High CCR4-I exhibits a strong correlation with high Treg infiltration. (**C**) Kaplan-Meier survival curves for OS and RFS of patients with HCC based on the Treg/CD8 ratio and CCR4 + CD73 + cell count. (Scale bar = 50 μm; *p < 0.05, **p < 0.01, ***p < 0.001)

We stained 873 HCC specimens with CD4, CD68, CD8, CD66b, and Foxp3 antibodies, revealing distinct immune cell types in the TME, where CD8 signifies cytotoxic T cells, CD66b is a surface marker of TAN, and Foxp3 represents Treg (Fig. 3A). We observed that high CCL17-T was correlated with increased Treg infiltration, reduced CD8 + T cell infiltration, and a higher Treg/CD8 ratio. This suggests that HCCs with high CCL17-T expression exhibited more Treg infiltration and a lack of CD8 + T-cell infiltration, implying an intimate relationship between CCL17-T and an anergic T-cell response against tumor cells (Fig. 3B). High CCL17-T expression also highly correlated with CD73 (Fig. 3B).

We proposed that CCL17, with its chemotactic function, might recruit TAN into the tumor stroma to form a suppressive barrier. Indeed, our analysis revealed that high CCL17 expression was associated with a significant increase in TAN infiltration (*p* < 0.01). Tumor tissues with high CCL17-T levels generally had high CCR4-I expression, suggesting that HCC cells might secrete CCL17 to recruit CCR4 + stroma cells (*p* < 0.001, Fig. 3B). Intriguingly, CCR4-I expression was found to be highly correlated with Treg infiltration (*r* = 0.4036, *p* < 0.001, Fig. 3B), leading us to speculate that CCR4 + CD73 + cells might be a special subset of Treg exerting an immunoinhibitory function.

Next, we further explored the relationship between CCL17, CCR4, and tumor angiogenesis in human HCC tissues. A CD34 antibody was used to stain vascular endothelial cells. Tumor tissues with high CCL17-T levels had significantly increased vascular endothelial cells genesis compared to those with low CCL17-T expression (Fig. 3B).

Subsequently, we divided the patients into two groups based on the ratio of Treg to CD8 and performed survival analysis. As illustrated in Fig. 3C, HCC patients with Treg/CD8 > 1 were associated with a higher probability of recurrence and significantly shortened survival time, which was corroborated in the validation cohort. We continued to explore the prognostic value of the specific group of cells, CCR4 + CD73+, defined earlier. Using 13 as the cutoff value, patients with higher CCR4 + CD73 + exhibited poor OS and RFS in both the training and validation cohorts.

### Univariate and multivariate analyses for OS and RFS in patients with HCC

Univariate Cox proportional regression analysis identified various risk factors for OS in the training cohort, including tumor number, size, differentiation, MVI, AFP, GGT, CD34, CD68, CD66b, Treg/CD8, CCR4-T, CCL17-T, CCL17-I, HHLA-2, CD73-T, and CCR4 + CD73 + cells. Factors associated with RFS were similar and included liver cirrhosis (all *P* < 0.050, Table 3). Multivariate analysis revealed that high CCL17-T maintained its prognostic significance for predicting both RFS [HR 1.774(1.274–2.470), *P* = 0.001] and OS [HR 1.818 (1.242–2.661), *P* = 0.002], along with tumor size, MVI, Treg/CD8, CCR4-T, HHLA-2-T, CD73-T, and CCR4 + CD73 + cells (Table 3).

In the validation cohort, tumor number, size, age, differentiation, MVI, GGT, CD34, CD66b, Treg/CD8, CCR4-T, CCL17-T, CCL17-I, HHLA-2, CD73-T, and CCR4 + CD73 + cells have been proved to be prognostic variables for both OS and RFS in univariate analysis (all *P* < 0.050, Table 4). Multivariate analysis revealed that high CCL17-T maintained its prognostic significance for predicting both RFS [HR 1.407 (1.117–1.771), *P* = 0.004] and OS [HR 1.633 (1.274–2.095), *P* < 0.001], along with tumor size, MVI, differentiation, CD34, Treg/CD8, CCR4-T, CCL17-I, HHLA-2-T, CD73-T, and CCR4 + CD73 + cells for OS, and age, tumor size, MVI, Treg/CD8, CCR4-T, CCL17-I, HHLA-2-T, CD73-T, and CCR4 + CD73 + cells for RFS (Table 4).

### Investigating the impact of CCL17, CCR4, and CCR4 + CD73 + cell profiles on clinical outcomes in HCC

**Fig. 4:**
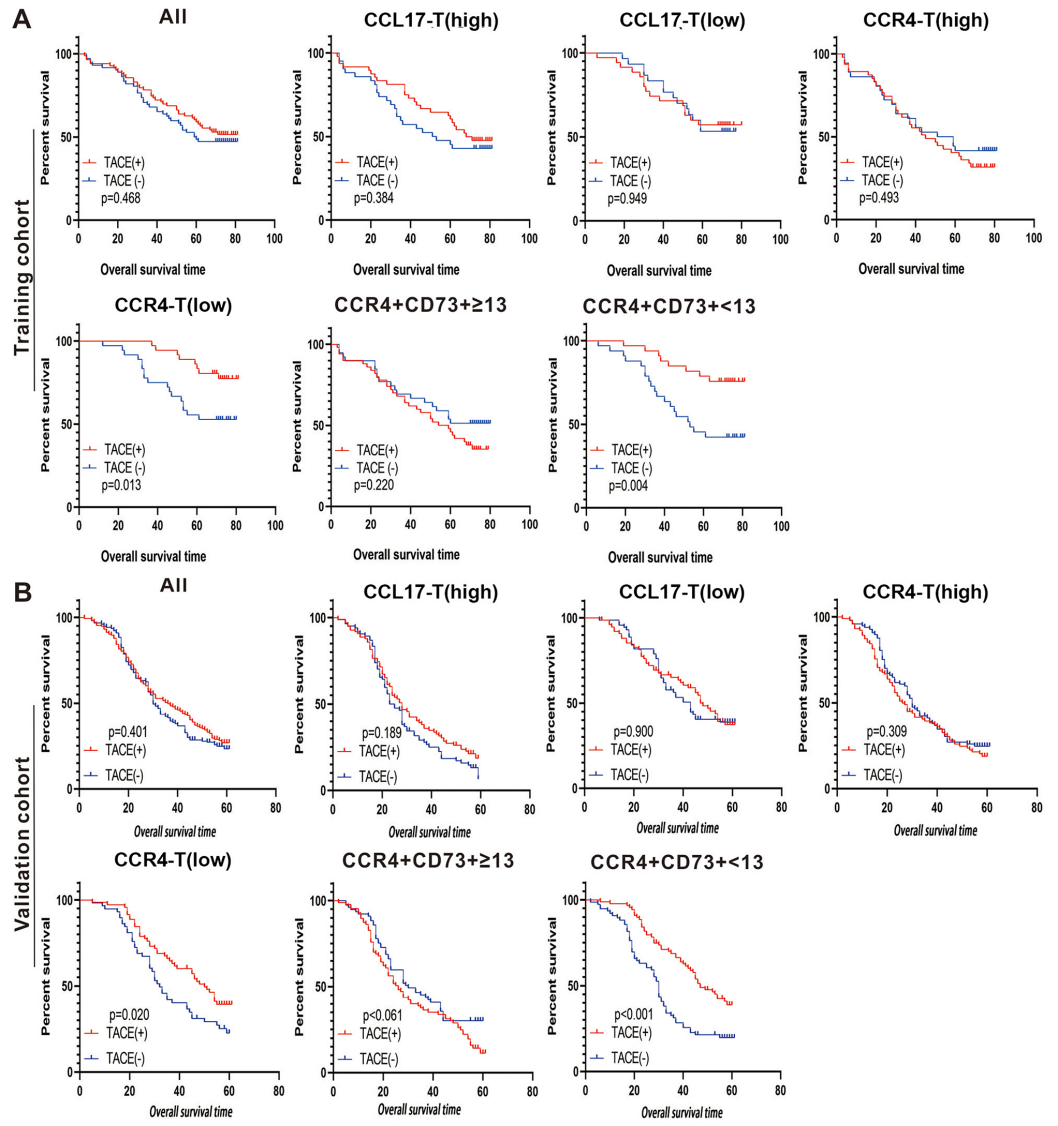
The relationship between CCL17-T, CCR4-T, and CCR4 + CD73 + Cells and the efficacy of TACE therapy in HCC with postoperative recurrence. Significantly improved prognosis associated with low CCR4 expression and CCR4 + CD73 + < 13 in HCC patients undergoing postoperative TACE after tumor recurrence, shown in both training cohort (**A**) and validation cohort (**B**)

In our study, 155 patients in the training cohort and 329 in the validation cohort experienced recurrence following radical surgical therapy. Figure 4 illustrates that there was no significant difference in OS between patients who underwent post-operative TACE and those who did not, across both cohorts. Further analysis revealed that HCC patients’ OS, regardless of their CCL17-T expression levels, was not markedly improved by post-operative TACE. A similar observation was made among patients with elevated CCR4-T levels. However, an intriguing pattern emerged within a specific subset of HCC patients; those with low CCR4-T expression significantly benefited from post-operative TACE in terms of OS. This beneficial effect was also observed in patients with low CCR4 + CD73 + expression, suggesting that TACE could offer considerable clinical advantages for HCC patients characterized by either low CCR4-T or CCR4 + CD73 + profiles. These findings imply that integrating TACE with targeted anti-CCR4 and anti-CD73 therapies might provide a novel and effective strategy for enhancing the survival of post-operative HCC patients. Considering the ongoing exploration of CCR4 and CD73 inhibitors in various clinical trials, a synergistic approach that includes TACE represents a promising avenue for advancing HCC post-operative treatment.

### Establishing new nomogram models for OS and RFS prediction

**Fig. 5:**
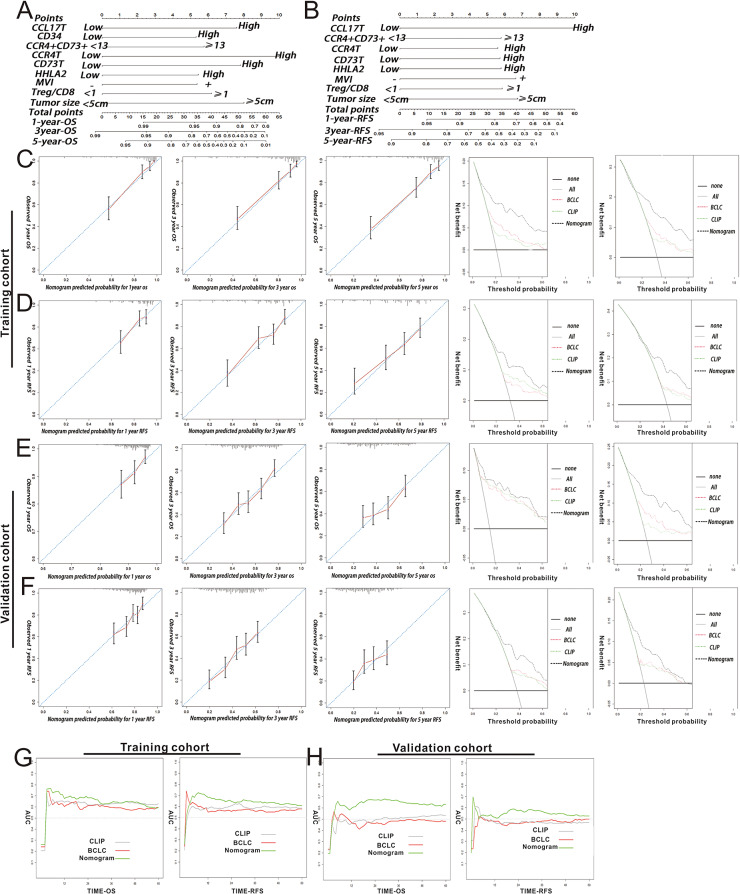
Nomogram Predictive Model for OS and RFS. (**A** and **B**) OS and RFS nomograms, respectively. (**C** and **E**) Calibration curves designed for predicting OS at 1 year, 3 years, 5 years. (**D** and **F**) Calibration curves designed for predicting RFS at 1 year, 3 years, 5 years. Nomogram-predicted possibility of survival marked on the x-axis; actual survival marked on the y-axis. (**G** and **H**) AUC curves for nomogram, BCLC, and CLIP. Dashed lines demonstrate the net benefit of the nomogram in each of the curves across a range of threshold probabilities. The horizontal solid black line represents the assumption that no patients would experience the event, and the solid gray line represents the assumption that all patients will die or relapse

We developed a nomogram to visually represent the predicted clinical outcome, drawing from multivariate regression analyses in both the training and validation cohorts. The multivariate Cox proportional regression model identified significant predictors as independent prognostic factors for OS: CCL17-T high, CD34 high, CCR4 + CD73 + ≥ 13, CCR4-T high, CD73-T high, HHLA2, MVI, Treg/CD8 ≥ 1, and tumor size ≥ 5 cm. These factors were then utilized to create a time-based nomogram model to forecast the survival probability of HCC patients at 1-, 3-, and 5- years post-surgery (Fig. 5A). The multivariate analysis also highlighted eight significant prognostic factors for RFS. A corresponding nomogram model was developed to predict 1-, 3-, and 5-year RFS (Fig. 5B).

The model’s calibration curve showcased a strong match between the predicted and actual survival rates at 1-, 3-, and 5- year for both cohorts. Additionally, a decision curve analysis was conducted to assess the clinical value and potential benefits of our model. The findings from this curve suggested that therapeutic strategies informed by our nomogram might enhance clinical outcomes (Fig. 5C and E). Predicted outcomes from the nomogram closely matched the real RFS in both cohorts. Notably, our new nomogram models displayed superior net benefits across a broader threshold probability spectrum (Fig. 5D and F). Furthermore, our OS and RFS nomograms demonstrated a higher area under the receiver operating characteristic curve when compared to the BCLC and CLIP (Cancer of the Liver Italian Program) stage systems in both cohorts (Fig. 5G-H).

## Discussion

In this study, we established a correlation between high expression levels of CCR4, CCL17, CD73, and HHLA2 in tumor tissues and the poor prognosis in HCC patients. HCC-derived CCL17 exhibited a robust relationship with CD73, present on both tumor and stroma cells, indicating its significant association with heightened CD73 expression in the TME. Interestingly, we recognized a stromal cell subtype, CCR4 + CD73 + co-expressing cells, which were closely associated with Tregs and predicted unfavorable outcomes. Patients with low CCR4 + CD73 + infiltration showed considerable improvement in OS after undergoing post-operative TACE. Moreover, patients with suppressed CCR4 expression also benefited from post-operative TACE. This data emphasizes the critical role of combining TACE with anti-CCR4 and anti-CD73 therapies in managing solid tumors. Additionally, we constructed novel nomogram models for predicting OS and RFS, enhancing comprehensive patient management and informed clinical decision-making.

Previously, CCL17 expression was only detected in peripheral blood neutrophils and TANs. In contrast, our findings revealed its expression in both tumor and stroma cells, as well as its receptor CCR4. Our results concur with previous report suggesting the stromal expression of CCL17 correlates with TANs (Zhou et al. 2016). Similarly, CCR4 expression in stroma was positively related to FOXP3 + Treg, known for inhibiting self-antigen immune responses (Maeda et al. 2019b). These observations align with those found in bladder and prostate cancer studies (Bergamaschi et al. 2020; Maeda et al. 2019b).

Our in-depth analysis indicated a strong association between CCL17 and the high Treg/CD8 ratio, indicative of an immunosuppressive environment (Bergamaschi et al. 2020; Mao et al. 2019). To substantiate our findings, we evaluated the relationship between CCL17 and various immune checkpoints, notably PD-L1, CD47, CD73, and HHLA2. Remarkably, CCL17 expression correlated robustly with CD73 but not with PD-L1, CD47, or HHLA2. With IF assays, we discovered that CCR4 and CD73 co-expressed on a unique subtype of stromal cells, which correlated highly with Treg and we termed it as CCR4 + CD73 + cells. Tregs co-express CD39, CD73 has been recognized for its pivotal role in immunosuppression and CD39-CD73-Adenosine axis is a crucial pathway in immune-related and malignant diseases, facilitating Treg induction and promoting immunosuppressive actions (Fu et al. 2016; Wang et al. 2021; Xia et al. 2023). Numerous studies have also highlighted the expression of both CCR4 and CD73 in Tregs (Huang et al. 2021; Plitas and Rudensky 2020). Building on these insights, we postulated that CCR4 + CD73 + might represent a unique Treg subtype. However, this proposition warrants further investigation. Our subgroup analysis reaffirmed that patients with elevated CCR4 + CD73 + infiltration exhibited worse OS and RFS than those with reduced infiltration, across both training and validation cohorts. This indicates that CCR4 + CD73 + Tregs may have substantial immunosuppressive capabilities, akin to other Treg types. Notably, when considering mechanisms to counteract cancer’s immune evasion tactics, CD73 emerged as more prevalent and bore a greater prognostic significance than PD-L1 and CD47 in HCC.

Recent therapies have placed considerable emphasis on anti-checkpoint treatments due to their efficacy in limiting Treg infiltration and tumor progression (Langhans et al. 2019). These include targeted therapies against significant checkpoints, such as mogamulizumab for CCR4 and CD73-siRNA delivery for CD73 (Azambuja et al. 2020; Maeda et al. 2019b). Given our findings and the extensive HCC specimen analysis, we propose that a combinatorial approach involving systematic anti-checkpoint therapy (targeting CCR4 and CD73) and TACE might offer a promising avenue for post-operative HCC management.

Although post-operative TACE is a common therapeutic strategy to prevent tumor recurrence, its benefits are limited to a subset of HCC patients (Dev et al. 2020; Wang et al. 2020a). We identified a direct correlation between CCR4 expression, CCR4 + CD73 + stromal cells in stroma, and adverse prognosis post-TACE. Hence, integrating TACE with anti-CCR4 and anti-CD73 therapies might enhance therapeutic efficacy and extend HCC patient survival significantly.

However, our study has limitations. Being a single-institution retrospective study, our nomogram models require external validation. Though our data is clinically-derived, in vitro functional experiments could augment its validity. Additionally, our study’s scope was limited to specific markers and checkpoints; further expansion is warranted.

In summary, our systematic examination of CCL17, CCR4, CD73, and HHLA2 offers a fresh perspective on refining HCC management and clinical decision-making. The identification of CCR4 + CD73 + cells as a novel stromal cell type associated with adverse prognosis provides crucial insights for post-operative TACE strategies, potentially revolutionizing HCC patient care.

## Electronic supplementary material

Below is the link to the electronic supplementary material.

Supplementary Material 1

## Data Availability

No datasets were generated or analysed during the current study.

## References

[CR1] Azambuja JH, Schuh RS, Michels LR (2020) Blockade of CD73 delays glioblastoma growth by modulating the immune environment. Cancer Immunol Immunother 69:1801–1812 32350590 10.1007/s00262-020-02569-w PMC11027675

[CR2] Baghban R et al (2020) Tumor microenvironment complexity and therapeutic implications at a glance cell. Communication Signal 18:59. 10.1186/s12964-020-0530-4 10.1186/s12964-020-0530-4 PMC7140346 32264958

[CR3] Bangaru S, Marrero JA, Singal AG (2020) Review article: new therapeutic interventions for advanced hepatocellular carcinoma. Aliment Pharmacol Ther 51:78–89 31747082 10.1111/apt.15573

[CR4] Bergamaschi C, Pandit H, Nagy BA (2020) Heterodimeric IL-15 delays tumor growth and promotes intratumoral CTL and dendritic cell accumulation by a cytokine network involving XCL1, IFN-γ, CXCL9 and CXCL10 J Immunother Cancer 8 10.1136/jitc-2020-000599 PMC7254133 32461349

[CR5] Bouchet M, Lainé A, Boyault C (2020) ERRα expression in bone metastases leads to an exacerbated Antitumor Immune. Response Cancer Res 80:2914–2926 32366476 10.1158/0008-5472.CAN-19-3584

[CR6] Da M, Chen L, Enk A, Ring S, Mahnke K (2022) The multifaceted actions of CD73 during Development and Suppressive Actions of Regulatory T. Cells Front Immunol 13:914799. 10.3389/fimmu.2022.914799 35711418 10.3389/fimmu.2022.914799 PMC9197450

[CR7] Dev A, Sood A, Choudhury K, Sr. S (2020) Paclitaxel nanocrystalline assemblies as a potential transcatheter arterial chemoembolization (TACE) candidate for unresectable hepatocellular carcinoma. Mater Sci Eng C Mater Biol Appl 107:110315 10.1016/j.msec.2019.110315 31761231

[CR8] Feng H et al (2022) Tumor Microenvironment in Hepatocellular Carcinoma: key players for Immunotherapy. J Hepatocell Carcinoma 9:1109–1125. 10.2147/JHC.S381764 36320666 10.2147/JHC.S381764 PMC9618253

[CR9] Fu YP, Yi Y, Cai XY (2016) Overexpression of interleukin-35 associates with hepatocellular carcinoma aggressiveness and recurrence after curative resection. Br J Cancer 114:767–776 27002937 10.1038/bjc.2016.47 PMC4984866

[CR10] Gan W, Yi Y, Fu Y (2018) Fibrinogen and C-reactive protein score is a prognostic index for patients with hepatocellular carcinoma undergoing curative resection: a prognostic nomogram study. J Cancer 9:148–156 29290780 10.7150/jca.22246 PMC5743722

[CR11] Gao Q et al (2007) Intratumoral balance of regulatory and cytotoxic T cells is associated with prognosis of hepatocellular carcinoma after resection. J Clin Oncol 25:2586–2593 17577038 10.1200/JCO.2006.09.4565

[CR12] Gao Y, Fan X, Li N (2020) CCL22 signaling contributes to sorafenib resistance in hepatitis B virus-associated hepatocellular carcinoma. Pharmacol Res 157:104800 32278046 10.1016/j.phrs.2020.104800

[CR13] Harvey JB, Phan LH, Villarreal OE, Bowser JL (2020) CD73’s potential as an Immunotherapy Target in Gastrointestinal. Cancers Front Immunol 11:508 32351498 10.3389/fimmu.2020.00508 PMC7174602

[CR14] Higuchi T, Matsuo K, Hashida Y (2019) Epstein-Barr virus-positive pyothorax-associated lymphoma expresses CCL17 and CCL22 chemokines that attract CCR4-expressing regulatory T. Cells Cancer Lett 453:184–192 30953706 10.1016/j.canlet.2019.03.053

[CR15] Huang L, Guo Y, Liu S, Wang H, Zhu J, Ou L, Xu X (2021) Targeting regulatory T cells for immunotherapy in melanoma. Mol Biomed 2:11. 10.1186/s43556-021-00038-z 34806028 10.1186/s43556-021-00038-z PMC8591697

[CR16] Ioannides CG, Whiteside TL (1993) T cell recognition of human tumors: implications for molecular immunotherapy of cancer. Clin Immunol Immunopathol 66:91–106 8453790 10.1006/clin.1993.1012

[CR17] Jing C-Y et al (2019) HHLA2 in intrahepatic cholangiocarcinoma: an immune checkpoint with prognostic significance and wider expression compared with PD-L1. J Immunother Cancer 7:1–11 30885276 10.1186/s40425-019-0554-8 PMC6421676

[CR18] Kotsari M, Dimopoulou V, Koskinas J, Armakolas A (2023) Immune System and Hepatocellular Carcinoma (HCC): New insights into HCC Progression International. J Mol Sci 24:11471 10.3390/ijms241411471 PMC10380581 37511228

[CR19] Langhans B, Nischalke HD, Krämer B (2019) Role of regulatory T cells and checkpoint inhibition in hepatocellular carcinoma. Cancer Immunol Immunother 68:2055–2066 31724091 10.1007/s00262-019-02427-4 PMC11028391

[CR20] Lim JB, Kim DK, Chung HW (2014) Clinical significance of serum thymus and activation-regulated chemokine in gastric cancer: potential as a serum biomarker. Cancer Sci 105:1327–1333 25154912 10.1111/cas.12505 PMC4462361

[CR21] Llovet JM et al (2021) Hepatocellular carcinoma. Nat Reviews Disease Primers 7:6. 10.1038/s41572-020-00240-3 33479224 10.1038/s41572-020-00240-3 PMC13537433

[CR22] Lupia M, Angiolini F, Bertalot G (2018) CD73 regulates stemness and epithelial-mesenchymal transition in ovarian Cancer-initiating cells. Stem Cell Rep 10:1412–1425 10.1016/j.stemcr.2018.02.009 PMC5998305 29551673

[CR23] Ma XL, Hu B, Tang WG (2020) CD73 sustained cancer-stem-cell traits by promoting SOX9 expression and stability in hepatocellular carcinoma. J Hematol Oncol 13:11 32024555 10.1186/s13045-020-0845-z PMC7003355

[CR24] Maeda S, Murakami K, Inoue A, Yonezawa T, Matsuki N (2019a) CCR4 Blockade Depletes Regulatory T Cells and prolongs survival in a canine model of bladder. Cancer Cancer Immunol Res 7:1175–1187. 10.1158/2326-6066.CIR-18-0751 31160277 10.1158/2326-6066.CIR-18-0751

[CR25] Maeda S, Murakami K, Inoue A, Yonezawa T, Matsuki N (2019b) CCR4 Blockade Depletes Regulatory T Cells and prolongs survival in a canine model of bladder Cancer. Cancer Immunol Res 7:1175–1187 31160277 10.1158/2326-6066.CIR-18-0751

[CR26] Mao W, Ghasemzadeh A, Freeman ZT (2019) Immunogenicity of prostate cancer is augmented by BET bromodomain inhibition. J Immunother Cancer 7:277 31653272 10.1186/s40425-019-0758-y PMC6814994

[CR27] Mishalian I et al (2014) Neutrophils recruit regulatory T-cells into tumors via secretion of CCL17–a new mechanism of impaired antitumor immunity. Int J Cancer 135:1178–1186. 10.1002/ijc.28770 24501019 10.1002/ijc.28770

[CR28] Plitas G, Rudensky AY (2020) Regulatory T cells in Cancer Annual. Rev Cancer Biology 4:459–477. 10.1146/annurev-cancerbio-030419-033428

[CR29] Sung H, Ferlay J, Siegel RL, Laversanne M, Soerjomataram I, Jemal A, Bray F (2021) Global Cancer statistics 2020: GLOBOCAN estimates of incidence and Mortality Worldwide for 36 cancers in 185 countries CA. Cancer J Clin 71:209–249. 10.3322/caac.21660 10.3322/caac.21660 33538338

[CR30] Tang A, Hallouch O, Chernyak V, Kamaya A, Sirlin CB (2018) Epidemiology of hepatocellular carcinoma: target. Popul Surveillance Diagnosis 43:13–25 10.1007/s00261-017-1209-1 28647765

[CR35] Wang Y, Weng X, Wang L (2018) HIC1 deletion promotes breast cancer progression by activating tumor cell/fibroblast crosstalk. J Clin Invest 128:5235–5250 30204129 10.1172/JCI99974 PMC6264654

[CR33] Wang S, Ge M, Cui J (2019) Diminished interaction between mutant NOTCH1 and the NuRD corepressor complex upregulates CCL17 in chronic. Lymphocytic Leuk Leuk 33:2951–2956 10.1038/s41375-019-0526-5 31341237

[CR31] Wang PX, Sun YF, Zhou KQ (2020a) Circulating tumor cells are an indicator for the administration of adjuvant transarterial chemoembolization in hepatocellular carcinoma: a single-center, retrospective, propensity-matched study. Clin Transl Med 10:137 32702202 10.1002/ctm2.137 PMC7418815

[CR34] Wang S, Li Y, Xing C (2020b) Tumor microenvironment in chemoresistance, metastasis and immunotherapy of pancreatic cancer. Am J Cancer Res 10:1937–1953 32774994 PMC7407356

[CR32] Wang S, Gao S, Zhou D, Qian X, Luan J, Lv X (2021) The role of the CD39-CD73-adenosine pathway in liver disease. J Cell Physiol 236:851–862 32648591 10.1002/jcp.29932

[CR36] Xia C, Yin S, To KKW, Fu L (2023) CD39/CD73/A2AR pathway and cancer immunotherapy. Mol Cancer 22:44. 10.1186/s12943-023-01733-x 36859386 10.1186/s12943-023-01733-x PMC9979453

[CR37] Yang JD, Hainaut P, Gores GJ, Amadou A, Plymoth A, Roberts LR (2019) A global view of hepatocellular carcinoma: trends, risk, prevention and management. Nat Rev Gastroenterol Hepatol 16:589–604 31439937 10.1038/s41575-019-0186-y PMC6813818

[CR38] Ye T et al (2022) Chemokine CCL17 affects local Immune infiltration characteristics and early prognosis value of Lung Adenocarcinoma Front. Cell Dev Biol 10:816927. 10.3389/fcell.2022.816927 10.3389/fcell.2022.816927 PMC8936957 35321241

[CR39] Yu M et al (2020) CD73 on cancer-associated fibroblasts enhanced by the A2B-mediated feedforward circuit enforces an immune checkpoint. Nat Commun 11:515. 10.1038/s41467-019-14060-x 31980601 10.1038/s41467-019-14060-x PMC6981126

[CR40] Zhang M et al (2018) Overexpression of interleukin-35 in intrahepatic cholangiocarcinoma is a prognostic indicator after curative resection. Cancer Sci 109:1195–1206 29446854 10.1111/cas.13535 PMC5891208

[CR42] Zheng R, Qu C, Zhang S (2018) Liver cancer incidence and mortality in China: temporal trends and projections to 2030. Chin J Cancer Res 30:571–579 30700925 10.21147/j.issn.1000-9604.2018.06.01 PMC6328503

[CR41] Zheng H et al (2023) Targeting tumor-associated macrophages in hepatocellular carcinoma: biology, strategy, and immunotherapy. Cell Death Discov 9:65. 10.1038/s41420-023-01356-7 36792608 10.1038/s41420-023-01356-7 PMC9931715

[CR44] Zhou SL, Zhou ZJ, Hu ZQ (2016) Tumor-Associated Neutrophils Recruit Macrophages and T-Regulatory cells to promote progression of Hepatocellular Carcinoma and Resistance to. Sorafenib Gastroenterol 150:1646–16581617 10.1053/j.gastro.2016.02.040 26924089

[CR43] Zhou J et al (2023) Guidelines for the diagnosis and treatment of primary Liver Cancer (2022 Edition) Liver Cancer. 1–40. 10.1159/000530495 10.1159/000530495 PMC10601883 37901768

