# Supplementary material for: Enhancing hepatocellular carcinoma management: prognostic value of integrated CCL17, CCR4, CD73, and HHLA2 expression analysis
Source: J Cancer Res Clin Oncol. 2024 Jun 25;150(6):325. doi: 10.1007/s00432-024-05832-0 (PMC11196339; doi:10.1007/s00432-024-05832-0)
Supplement: Supplementary file 1 — Supplementary Material 1 [file 432_2024_5832_MOESM1_ESM.doc]

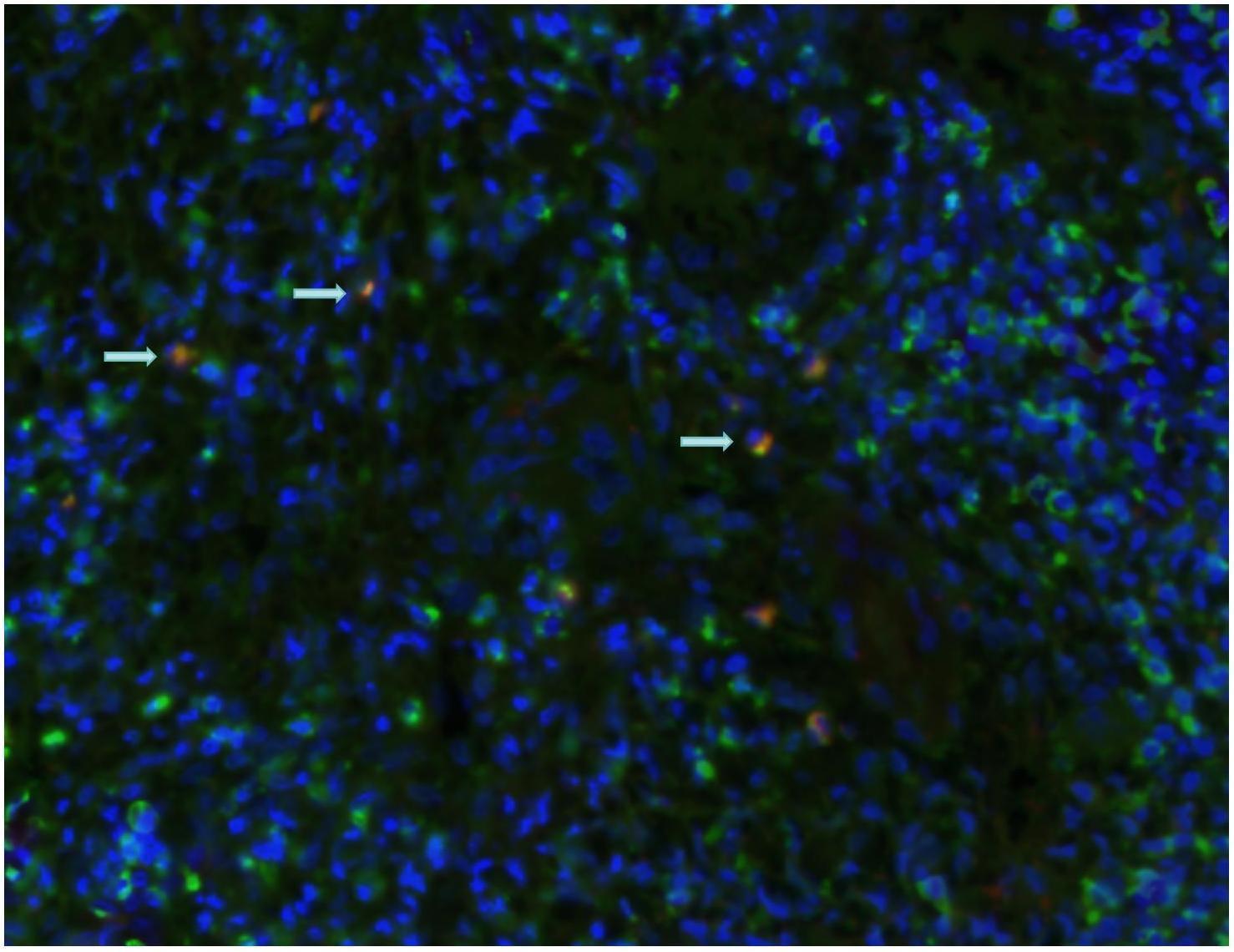


Fig S1. Double staining of CCR4 and CD73 in tumor stromal cells. The blue fluorescence by DAPI highlights the cell nucleus, while the green fluorescence represents CCR4 staining, and the red fluorescence represents CD73 staining. Arrows indicate cells displaying both green and red staining, indicating CCR4+CD73+ cells.
